# Targeted radionuclide therapy against GARP expressing T regulatory cells after tumour priming with external beam radiotherapy in a murine syngeneic model

**DOI:** 10.1016/j.heliyon.2024.e39543

**Published:** 2024-10-18

**Authors:** Pierre-Simon Bellaye, Alexandre MM. Dias, Jean-Marc Vrigneaud, Alexanne Bouchard, Mathieu Moreau, Camille Petitot, Claire Bernhard, Michael Claron, Lisa Froidurot, Véronique Morgand, Mélanie Guillemin, Marie Monterrat, Céline Mirjolet, Carmen Garrido, Evelyne Kohli, Bertrand Collin

**Affiliations:** a Centre George-François Leclerc, Service de Médecine Nucléaire, IMATHERA UMS INSERM BioSanD US58, 1 rue du Professeur Marion, 21079, Dijon, France; b UMR INSERM/uB/AGROSUP 1231, Labex LipSTIC, Faculty of Health Sciences, Université de Bourgogne Franche-Comté, 21079, Dijon, France; c Institut de Chimie Moléculaire de l’Université de Bourgogne, UMR CNRS/uB 6302, Université de Bourgogne Franche-Comté, 21079, Dijon, France; d University Hospital Centre François Mitterrand, 21000, Dijon, France

**Keywords:** GARP, Targeted radionuclide therapy, T regulatory cells, External beam radiotherapy, SPECT imaging

## Abstract

**Purpose:**

Radiation therapy (RT) exerts its anti-tumour efficacy by inducing direct damage to cancer cells but also through modification of the tumour microenvironment (TME) by inducing immunogenic antitumor response. Conversely, RT also promotes an immunosuppressive TME notably through the recruitment of regulatory T cells (Tregs). Glycoprotein A repetitions predominant (GARP), a transmembrane protein highly expressed by activated Tregs, plays a key role in the activation of TGF-β and thus promotes the immunosuppressive action of Tregs. The development of a theranostic approach targeting GARP combining imaging and targeted radionuclide therapy (TRT) was carried out.

**Methods:**

A preclinical model of 4T1 triple negative breast tumour-bearing BALB/c mice was used to show that GARP expression is increased after external beam radiation in the TME of our cancer model. We generated a theranostic probe through the bioconjugation of the chelating agent DOTAGA onto an anti-GARP monoclonal antibody. The bioconjugation with DOTAGA allows the radiolabelling of the DOTAGA-GARP conjugate with both Indium-111 for SPECT imaging and Lutetium-177 for TRT purposes.

**Results:**

We demonstrate that GARP expression is increased following RT *in vivo* and can be specifically detected and quantified using *in vivo* SPECT imaging with [^111^In]In-DOTAGA-GARP. In addition, ^177^Lu-DOTAGA-GARP limits tumour growth in our cancer model.

**Conclusion:**

This theranostic strategy may allow for the personalization of cancer treatments by early detection of activated Tregs infiltration following RT and identification of patients likely to respond to Tregs-targeted therapy *via* TRT.

## 1 Introduction

More than half of cancer patients are treated with radiation therapy (RT) at some stage in their care pathway [1], making RT one of the most widely used treatment for cancer [2]. RT exerts its anti-tumour efficacy by inducing direct damage to cancer cells, such as double-strand DNA breaks, but also through modification of the tumour microenvironment (TME) by inducing a local and systemic immune response [[3], [4], [5]], notably by activating CD8^+^ T cells [6]. Conversely, at later stages RT also promotes the recruitment and activation of regulatory T cells (Tregs) [7,8] and pro-tumour M2-like macrophages [9], hence favouring an immunosuppressive TME promoting tumour growth and resistance to RT.

Tregs are immunosuppressive cells which have been shown to inhibit the anti-tumour immune response and promote tumour progression [10]. Moreover, the number of tumour-infiltrating Tregs is significantly increased in many cancers such as breast, lung and gastrointestinal tumours and represent a marker of poor prognosis and treatment failure in many patients [11]. Due to their immunosuppressive activity, Tregs represent a prime target for immunotherapies. However, the lack of accessible specific markers for Tregs has limited their use for therapeutic and diagnostic purposes. To date, markers for Tregs are mainly transcription factors such as FoxP3 and Helios but their nuclear localization limits their therapeutic targeting as well as the detection of Tregs by *in vivo* imaging.

The immunosuppressive action of Tregs is largely mediated by Transforming Growth Factor (TGF)-β [12], a cytokine that is paramount in the process of the progression of advanced tumours. In addition to its role in invasion and metastasis [13,14], TGF-β promotes tumour evasion from the immune system by inhibiting the anti-tumour functions of immune cells such as CD8^+^ T cells and NK cells [15,16]. Moreover, the impact of TGF-β expression in the context of induced resistance to RT has already been described [17]. In this context, Glycoprotein A repetitions predominant (GARP encoded by *LRRC32* gene) is a transmembrane protein that is highly expressed by activated Tregs but not by other T cell subtypes [[18], [19], [20]]. GARP plays a key role in the activation of TGF-β and thus promotes the immunosuppressive phenotype of Tregs and in maintaining Treg-mediated peripheral tolerance [[21], [22], [23]]. Indeed, GARP binds the latent TGF-β complex and allows its activation by association of this complex with integrin-αVβ8 [24]. Thus, activated Tregs expressing GARP constitute a "reservoir" of TGF-β which, once activated, releases its immunosuppressive effects. Besides Tregs, GARP can also be found at the surface of cancer cells and thus also supports cancer cell growth and dissemination *via* the TGF-β axis [25]. In addition, GARP is also able to shut down anti-tumour T cell mediated immune responses [25]. Consequently, GARP appears to be a membrane marker of activated Tregs and cancer cells within tumours, accessible for *in vivo* imaging and therapeutic targeting [[26], [27], [28]]. Triple negative breast cancer (TNBC) is an aggressive cancer, associated with a poor prognosis, a high rate of metastasis and a lack of therapeutic targets [29]. The infiltration of GARP-expressing cells, their involvement in tumour growth and their immunosuppressive role on the TME have recently been shown in TNBC [26,30]. Moreover, external radiotherapy is a recommended therapeutic strategy for TNBC and therefore could increase Tregs [31]. The identification of a therapeutic target and associated biomarker in TNBC could improve the outcome and management of patients currently ineligible for targeted therapies.

Nuclear molecular imaging techniques have become indispensable in oncology, in particular thanks to [^18^F]-FDG PET, which now has a crucial impact on diagnosis, staging and monitoring of therapeutic efficacy. However, it is now possible to go further by designing a more specific radiopharmaceutical (*i.e*. a vector combined with a radionuclide) either for nuclear imaging (*e.g.* Indium-111 for SPECT) or for targeted radionuclide therapy (TRT, *e.g.* lutetium-177). This strategy allows to confer so-called theranostic properties (*i.e.* therapeutic and diagnostic) to a given molecule, by changing the radionuclide according to the desired application. TRT allows for selective and continuous irradiation of tumours with a low dose rate. Compared to almost all other systemic cancer treatment options, TRT has shown efficacy with minimal toxicity. Based on these elements, our work describes the development of a theranostic approach targeting GARP which may allow for the personalization of cancer treatments by early detection of Tregs infiltration following RT and identification of patients likely to respond to Tregs-targeted therapy *via* TRT.

## 2 Material and methods

### 2.1 Cell lines and reagents

The mouse triple-negative 4T1 breast cancer cell line was obtained from the American Type Culture Collection (ATCC® CRL-2539™, ATCC, Manassas, VA, USA) and was cultured in RPMI 1640 medium with 10 % foetal bovine serum (FBS) at 37 °C, 5 % CO_2_.

### 2.2 Triple-negative breast cancer (TNBC) model

All animal studies were performed in compliance with preclinical ethical guidelines and approved by the local ethical committee (C2EA Grand Campus, project #13445, Dijon, France). A syngeneic orthotopic murine model of TNBC was used. A total of 5x10^4^ 4T1 cells were administered using a 27G needle (total volume of 50 μL in RPMI 1640 medium without foetal bovine serum FBS) into the mammary fat pad of immunocompetent 8-week-old female BALB/c mice (Charles River, Ecully, France). All the administrations of cells were performed under anaesthesia by isoflurane 2 %. All animals were kept under pathogen-free conditions at 22 °C, 33 % relative humidity (RH), with a controlled 12 h day-night cycle, and had free access to standard diet and mineral water. Tumour volume (TV) was monitored daily with a calliper and calculated as TV = (x∗y^2^)/2, where x is the largest diameter and y is the corresponding orthogonal diameter.

Flow cytometry.

### 2.3 *In vitro* detection of GARP expression in 4T1 cells

4T1 cells were cultured in 6-well plates (500,000 cells per well) in 10 % foetal bovine serum (FBS) RPMI 1640 medium. Cells were irradiated using a small animal irradiator (SARRP, Xstrahl, UK), with 225 kV energy X-ray photons, 10 × 10 cm fix collimator and a dose rate of 1 Gy/min at the dose of 8Gy. 48h post irradiation, 4T1 cells were washed and saturated prior staining for 20 min with GARP monoclonal antibody (YGIC86, APC, Thermofisher 17-9891-82) or isotype control (APC, Rat IgG2a k Isotype Control 553932). Flow cytometry acquisition was performed on a Cytoflex 13C cytometer (Beckman Coulter). FlowJo™ v10.8.1 Software (BD Life Sciences) was used for analysis. Gating strategy is presented in Supplemental Fig. 1.

### 2.4 *Ex vivo* detection of GARP in 4T1 tumour bearing mice

Fourteen days after injection of 4T1 cells, mice were randomized into two groups receiving or not a single-fraction beam photon irradiation (8 Gy). Tumour irradiations were achieved using a small animal irradiator (SARRP, Xstrahl, UK) with 225 kV X-ray at a dose rate of 3.1 Gy/min to deliver 8 Gy in one fraction. Balistic used two opposite beams focusing on the tumour and totally avoiding the mouse's healthy organs, as described previously [32]. The modulation of GARP expression by 8 Gy irradiation was evaluated by flow cytometry 7 days after treatment. After dissection, tumours were mechanically and enzymatically dissociated using a mouse tumour dissociation kit according to manufacturer's recommendation (130-096-730 Miltenyi Biotech). Tumours were cut into small pieces and transferred into gentleMACS C tubes containing the enzyme mix. The dissociation was performed using the gentleMACS Octo Dissociator with heaters and with the mouse tumour dissociation 37C_m_TDK_2 program. Samples were homogenized before being applied to a MACS SmartStrainer 70 μM (130-110-916, Miltenyi Biotec) placed on a 15 mL tube. Filters were washed with 10 mL serum-free RPMI and then centrifuged at 300 g for 7 min. After complete aspiration of the supernatant, tumour cell suspensions were resuspended in RPMI and counted with trypan blue. Tumour cell suspension (10^6^ cells) was stained with specific antibodies according to manufacturer's recommendation (antibody details are presented in additional file) during 15 min at 4 °C in the dark. To analyze Tregs, the tumour cell suspension was stained with the FOXP3 staining buffer set (130-093-142 Miltenyi Biotech). Viability dye (L34964, ThermoFisher Scientific) was used to identify live cells. Flow cytometry acquisition was performed on Cytoflex 13C cytometer (Beckman Coulter). FlowJo™ v10.8.1 Software (BD Life Sciences) was used for analysis. Gating strategy is presented in Supplemental Fig. 2.

### 2.5 Immunofluorescence

Mouse tumour 4T1 biopsy sections were successively immersed in xylene (3x, 5 min), ethanol 100 % (2x, 5 min), ethanol 90 % (1x, 3 min), and 70 % (1x,3 min) before being rehydrated in a PBS-Tween 0.1 % (PBS-T) buffer (2 × 3 min). Antigen unmasking was achieved by heating at 95 °C in a 10 mM citrate buffer for 30 min and slides were cooled for 30 min at room temperature (RT). Saturation was performed by 1h incubation with a PBS-T, 8 % BSA solution. The tissues were then stained with a rabbit polyclonal antibody directed against GARP (1/48; Abcam, ab231214, Cambridge, UK) overnight at 4 C.

The tissues were then washed 3 times with PBS-T and incubated for 1h at RT with an anti-rabbit Alexa 568 (Abcam, ab175470, Cambridge, UK). Slides were then washed 3 times and mounted with a cover slide using a mounting medium with DAPI (Thermo Fisher Scientific, Waltham, MA, USA) for nuclear staining. Finally, slides were observed using a fluorescence microscope (Zeiss, Oberkochen, Germany), analysed with ZEN® software (Zeiss, Oberkochen, Germany) and quantified by the open-source program (ImageJ/Fiji software, NIH, Bethesda, MD, USA). Briefly, each colour channel (DAPI, blue and GARP, red) was separated. The area represented by GARP staining was quantified by the ImageJ software and expressed as the percentage of total tumour section area.

### 2.6 DOTAGA bioconjugation of GARP monoclonal antibody

The conjugation of the GARP monoclonal antibody (ALX-804-867, Plato-1, Enzo life sciences) with the bifunctional chelating agent DOTAGA, was carried out in collaboration with the ICMUB (Institut de Chimie Moléculaire de l’Université de Bourgogne – ICMUB UMR CNRS 6302, Dijon, France) [33]. Briefly, the anti-GARP mAb in PBS pH 7.4 was mixed with the appropriate volume of a 20 mM solution of DOTAGA anhydride in anhydrous DMSO (1.2, 2.3 and 3.5 μL for 10, 20 and 30 equivalents, respectively). The mixture was homogenized and left to incubate at 25 °C for 30 min. The reaction mixture were transferred into 2 mL Amicon® ultrafiltration devices (cut-off 50 kDa) and washed four times with PBS pH 7,4. The concentrations were determined by UV–Visible spectrophotometry at 280 nm. The degrees of labelling (DOL) were determined by mass spectrometry (MALDI-TOF) and were 2.5, 4.1 and 4.7 for 10, 20 and 30 equivalents, respectively (Supplemental Fig. 3).

For the remainder of the manuscript, this monoclonal antibody-based probe will be referred to as the DOTAGA-GARP probe.

### 2.7 Determination of the dissociation constant (Kd) by micro-scale thermophoresis

Three batches of probes (10; 20; 30 eq GARP-DOTAGA) prepared with different bifunctional chelating agent (DOTAGA)/anti-GARP antibody ratio were assayed (10 eq = 10 DOTAGA for 1 GARP antibody, 20 eq = 20 DOTAGA for 1 GARP antibody and 30 eq = 30 DOTAGA for 1 GARP antibody). The recombinant GARP protein (6055-LR-050, Bio-Techne, Minneapolis, USA) was labelled using the Protein Labelling Kit RED-Tris-NTA (MO-L018, NanoTemper Technologies, München, Germany). The labelling reaction was performed according to the manufacturer's instructions in the supplied labelling buffer, applying a concentration of 1 μM at room temperature for 30 min in the dark. Unreacted dye was removed by centrifugation. The degree of labelling was determined using UV/VIS spectrophotometry at 650 and 280 nm. A degree of labelling of 0.8 was typically achieved. The labelled recombinant GARP protein was adjusted to 200 nM with PBS buffer supplemented with 0.05 % Tween 20. The ligand, an anti-GARP antibody (ALX-804-867, Plato-1, Enzo life sciences) or our three DOTAGA-GARP probes was dissolved in PBS buffer supplemented with 0.05 % Tween 20 and a series of 16 1:1 dilution were prepared using the same buffer. For the measurement, each ligand dilution was mixed with one volume of labelled protein GARP, which led to a final concentration of protein GARP of 100 nM and final ligand concentrations ranging from 152.5 pM to 5 μM. After 10 min of incubation followed by centrifugation at 10,000×*g* for 10 min, the samples were loaded into Monolith NT.115 Capillaries (NanoTemper Technologies, München, Germany). MST was measured using a Monolith NT.115 instrument (NanoTemper Technologies, München, Germany) at an ambient temperature of 25.5 °C. Instrument parameters were adjusted to 100 % LED power and medium MST power. Data of three independently pipetted measurements were analysed (MO.Affinity Analysis software version 2.3, NanoTemper Technologies) using the signal from an MST and a time of 1s.

### 2.8 Determination of DOTAGA-GARP probe affinity

Affinity study was performed on 4T1 cells expressing GARP. 4T1 cells were seeded in 96-well plates (400,000 cells per well). The medium was removed by centrifugation (300 G, 5 min, 4 °C), followed by 2 washes with PBS (150 μL per well, 3 min). Cells were then saturated with a 3 % PBS-BSA solution (150 μL per well) for 45 min at 4 °C. After centrifugation, an increasing amount of radiolabelled antibody was added to each well to obtain the desired concentrations (from 0.05 to 50 nM), with or without an excess of unlabelled DOTAGA-GARP probe (10 μM). The plate was incubated for 2h at 4 °C. Three washes with PBS were performed. Cells were resuspended in 150 μL PBS and the total contents of each well collected in a 5 mL tube for gamma counting (Wizard® 1480, PerkinElmer, Villebon-sur-Yvette, France).

### 2.9 Radiolabelling of the DOTAGA-GARP probe

Radiolabelling of the DOTAGA-GARP probe was carried out using indium 111 or lutetium 177. A given volume of indium 111 or lutetium 177 was transferred to a LoBind® microtube (Eppendorf, Germany) with the DOTAGA-GARP probe to obtain an activity of 15 MBq for 25 μg GARP-DOTAGA probe per mouse for the [^111^In]In-DOTAGA-GARP probe or an activity of 3.7 MBq and 11.7 Mbq for 25 μg per mouse for the [^177^Lu]-DOTAGA-GARP probe. A quantity of 1 M AcONH4 buffer equal to = 1/10th of the volume of Indium 111 or lutetium 177 was added. The microtube was then placed in a thermomixer at 37 °C for 1 h, shaking at 1000 rpm. 0.1 M EDTA was added to the mixture (1/10th of total volume) and 1 μL of the resulting solution was spotted on an Instant Thin Layer Chromatography (ITLC-SG) strip and eluted in 0.1 M sodium citrate pH = 5, to determine the radiolabelling yield (*i.e.* radiochemical purity). The radiolabelled product was purified, buffer exchanged and concentrated by tangential flow filtration in an Amicon® (MWCO 30 kDa, 2 mL). The purified radiolabelled antibody was transferred to a new microtube and diluted with 0.9 % NaCl to obtain 25 μg of DOTAGA-GARP in 100 μL (approx. 15 MBq) for [^111^In]In-DOTAGA-GARP probe or to obtain 25 μg of antibody in 100 μL at the activity of 3.7 or 11.7 MBq for the [^177^Lu]Lu-DOTAGA-GARP probe prior to injection.

### 2.10 *In vivo* experiments

Our study exclusively examined female mice because the disease modelled (*i.e.* TNBC) is much more common in women.

**For the [**^**111**^**In]In-GARP-DOTAGA probe**, 4T1 tumour-bearing mice were randomized into two groups 14 days post-implantation, one group (n = 18) receiving external irradiation (8Gy) as described above, and a control group (n = 17) not irradiated. Six days post-irradiation, mice received an intravenous (i.v.) dose of 25 μg/15 MBq/100 μL of [^111^In]In-DOTAGA-GARP per mouse or an i.v. dose of 25 μg/15 MBq/100 μL of [^111^In]In-DOTAGA-GARP plus an excess (100x) of unlabelled DOTAGA-GARP per mouse (blocking group). SPECT/CT imaging was performed 24h and 48h post-injection. Mice were euthanized after the last imaging. Organs were collected for gamma counting (Wizard® 1480, PerkinElmer, Villebon-sur-Yvette, France).

**For the [**^**177**^**Lu]Lu-GARP-DOTAGA probe**, 4T1 tumour-bearing mice were randomized into 5 groups of 12 mice at 14 days post-implantation, mice in each group were subdivided in two groups receiving (IR 8 Gy n = 6) or not (NT n = 6) external irradiation at the dose of 8Gy. 7 days post-irradiation, mice received: one i.v. dose of 25 μg/3.7 MBq/100 μL of [^177^Lu]Lu-DOTAGA-GARP per mouse (G1 = 6 NT and G2 = 6 IR 8Gy), one i.v. dose of 25 μg/11.7 MBq/100 μL of [^177^Lu]Lu-DOTAGA-GARP per mouse (G3 = 6 NT and G4 = 6 IR 8Gy), one i.v. dose of 25 μg/11.7 MBq/100 μL of ^177^Lu-DOTAGA-irrelevant antibody (BE0083, Bio X Cell, USA) per mouse (G5 = 3 NT and G6 = 3 IR 8Gy), one i.v. dose of 25 μg/100 μL of non-radiolabelled DOTAGA-GARP per mouse (G7 = 6 NT and G8 = 6 IR 8Gy), one i.v. dose of 100 μL PBS per mouse(G9 = 6 NT and G10 = 6 IR 8Gy).

Imaging was performed at 48 h post-injection on groups 2 and 3. Tumour volumes and blood counts were measured three times a week. Mice were euthanized when tumour volumes exceeded ethical limits (*i.e.* > 1500 mm^3^).

### 2.11 Image processing

All SPECT and CT images were obtained using VivoQuant™ software (Invicro, Needham, USA). Each image was interpreted visually and the 3D regions of interest (ROIs) corresponding to the tumours were delineated manually in order to measure their radioactive content. The dose injected into each mouse was measured at the time of injection in MBq. The radioactive content of the tumour was expressed in MBq, then converted into percentage of the injected dose per gram of tissue (% ID/g). All images were decay-corrected for quantification.

### 2.12 Statistical analysis

For all experiments, statistical analyses were performed by non-parametric Mann-Whitney t-tests to compare two groups and 2 way ANOVA multiple comparisons Tukey tests to compare multiple group using GraphPad Prism 8.0 (GraphPad software, San Diego, CA, USA). All results are represented as the median with an interquartile range. The level of significance was set at p < 0.05.

## 3 Results

### 3.1 GARP expression is increased after external beam radiation therapy in the 4T1 tumour model

**Fig. 1:**
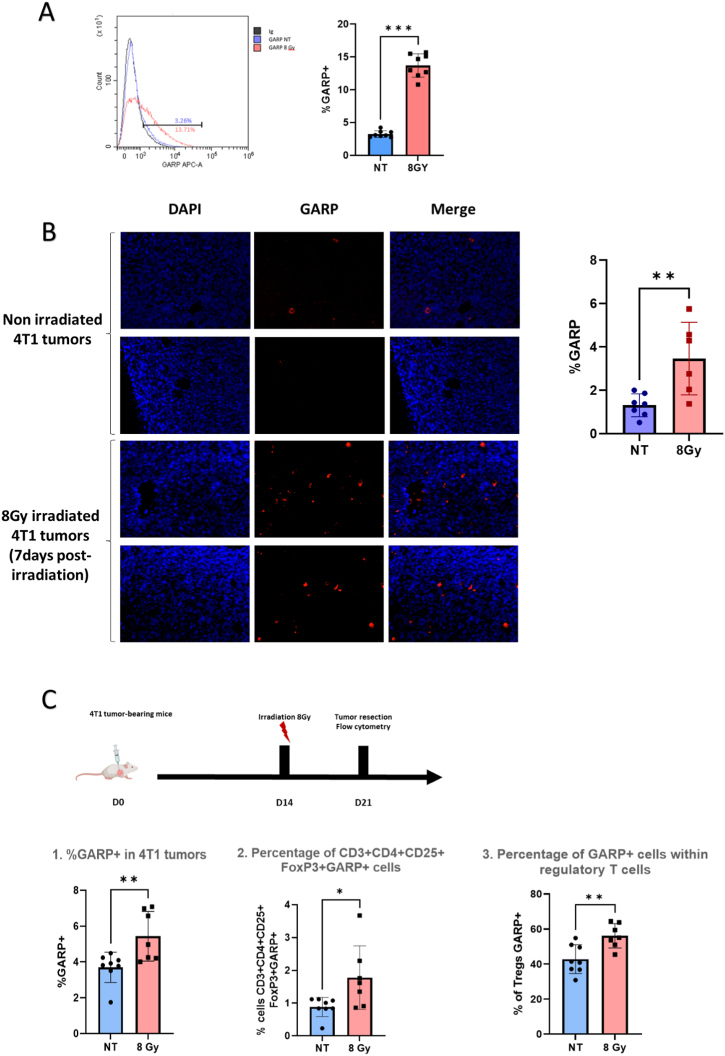
GARP expression with or without external beam radiation therapy in 4T1 tumour model. *A. In vitro detection of membrane GARP expression in 4T1 cells with or without external beam radiation therapy at the dose of 8 Gy (48h post irradiation) by flow cytometry. Results are presented as the median with the interquartile range, p=0.0002. B. Representative immunostaining and quantification of GARP (red) in 4T1 tumour from mice 7 days after 8 Gy irradiation (n=6) or not (n=7). Results are presented as the median with the interquartile range, p=0.0082. C. Schematic representation of the experimental design: ex vivo detection of GARP expression in 4T1 tumours by flow cytometry. 14 days after 4T1 cells injection mice were divided into two groups: control or 8 Gy irradiation. 7 days after irradiation, flow cytometry was performed on dissociated tumours. 1. Percentage of GARP + cells in alive cells in tumours (p=0.0093). 2. Percentage of CD3+CD4+CD25+FoxP3 + cells expressing GARP in the tumour (p=0.018). 3. Percentage of GARP + cells within CD3+CD4+CD25+FoxP3+ regulatory T cells (p=0.0067)*.

GARP is known to be expressed by cancer cells and activated Tregs (24). Flow cytometry demonstrated a low membrane expression of GARP at the 4T1 cell surface (3.26 % ± 0.18 %). However, upon irradiation at the dose of 8 Gy, the membrane expression of GARP was increased compared to non-irradiated 4T1 cells at 48h (13.71 % ± 0.63 % *vs* 3.26 % ± 0.18 % respectively, ∗∗∗p = 0.0002, Fig. 1A).

Total GARP expression was then analysed *ex vivo* in 4T1 tumour biopsies by immunofluorescence. The results showed a significant increase in GARP^+^ cells in the tumour microenvironment 7 days post-irradiation (8 Gy single fraction) compared to non-irradiated mice (3.47 % ± 0.68 % *vs* 1.31 % ± 0.53 % of nucleated cells in the tumour, respectively, ∗∗p = 0.0082, Fig. 1B).

These results were confirmed by flow cytometry showing an increase in the global expression of GARP in irradiated 4T1 tumour-bearing mice 7 days post-irradiation (5.44 % ± 0.52 % *vs* 3.70 % ± 0.29 % of cells in the tumour, ∗∗p = 0.0093, Fig. 1C). As GARP is known to be expressed at the surface of Tregs, the quantity of GARP^+^ Tregs cells (CD3^+^, CD4^+^, CD25^+^, FOXP3^+^, and GARP^+^) was also analysed. In non-irradiated mice, GARP^+^ Tregs infiltration in our 4T1 model was 0.88 % ± 0.10 % while, after irradiation, the GARP^+^ Tregs population was significantly increased in tumours (1.78 % ± 0.37 %, ∗p = 0.0180). Furthermore, the proportion of Tregs expressing GARP was also increased upon irradiation (56.16 % ± 2.61 % *vs* 42.86 % ± 2.90 %, ∗∗p = 0.0067, Fig. 1C).

Altogether, these results demonstrate an increase in GARP expression by activated Tregs as well as by tumour cells in the tumour microenvironment after a single radiation therapy session. To confirm these results, we developed a probe to monitor GARP expression *in vivo* in our TNBC animal model.

### 3.2 Design and *in vitro* validation of a GARP-targeted probe

**Fig. 2:**
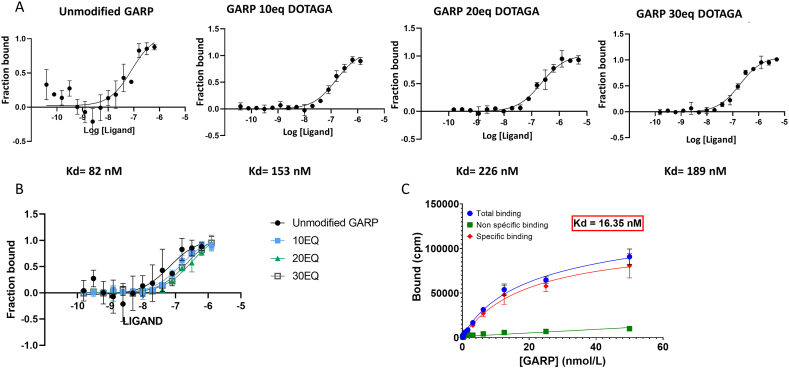
**Bioconjugation of DOTAGA on anti-GARP antibody: binding assays**. A/B. Saturation curve of the recombinant GARP protein with the unmodified antibody GARP; the DOTAGA-GARP 10eq (Kd = 153 nM); 20eq (Kd = 226 nM) and 30eq (Kd = 189 nM). C. Radioligand saturation binding to 4T1 cells of [^111^In]In-DOTAGA-GARP in the absence (total binding) or presence (non-specific binding) of excess (10 μM) unlabelled DOTAGA-GARP antibody. Specific binding was calculated by subtraction of non-specific binding from total binding.

The GARP monoclonal antibody ALX-804-867 was bioconjugated with the chelating agent DOTAGA (DOTAGA-GARP), able to complex indium-111 (^111^In) or lutetium-177 (^177^Lu). To analyze whether this modification had an impact on the affinity of the antibody for GARP, different batches of DOTAGA-GARP probes were prepared with different DOLs (degree of labelling) of DOTAGA per antibody, enabling the most suitable conjugation condition to be determined. The Kd of these three probes (10 eq, 20 eq and 30 eq) was assessed using microscale thermophoresis. Kd measurements of the binding between the recombinant GARP protein and the original non-bioconjugated anti-GARP antibody fragment revealed an affinity of around 82 nM. Bioconjugation with different degrees of labelling (10 eq, 20 eq and 30 eq) induced a slight decrease of affinity for GARP which remained however in the 100 nM range as for the non-bioconjugated antibody. As the affinity of the different probes was of the same order of magnitude, the GARP 30 eq DOTAGA probe was selected for future experiments, as it was expected to provide a higher yield of radionuclide incorporation (Fig. 2A).

In addition, the affinity of the selected DOTAGA-GARP probe was further evaluated with a radioligand saturation binding assay after radiolabelling with ^111^In. Total binding was determined by incubating a fixed quantity of 4T1 cells expressing GARP (4T1 cells irradiated at the dose of 8 Gy) with an increasing quantity [^111^In]In-DOTAGA-GARP). Non-specific antibody binding was determined using the same protocol as for total binding, except that a large excess of unlabelled DOTAGA-GARP was added to the medium. The specific binding curve was calculated as the difference between the total and non-specific binding curves and gave a Kd of 16.35 nM showing a compatibility for *in vivo* imaging experiments.

### 3.3 External beam radiation therapy increases [^111^In]-GARP-DOTAGA uptake *in vivo* by SPECT/CT imaging

**Fig. 3:**
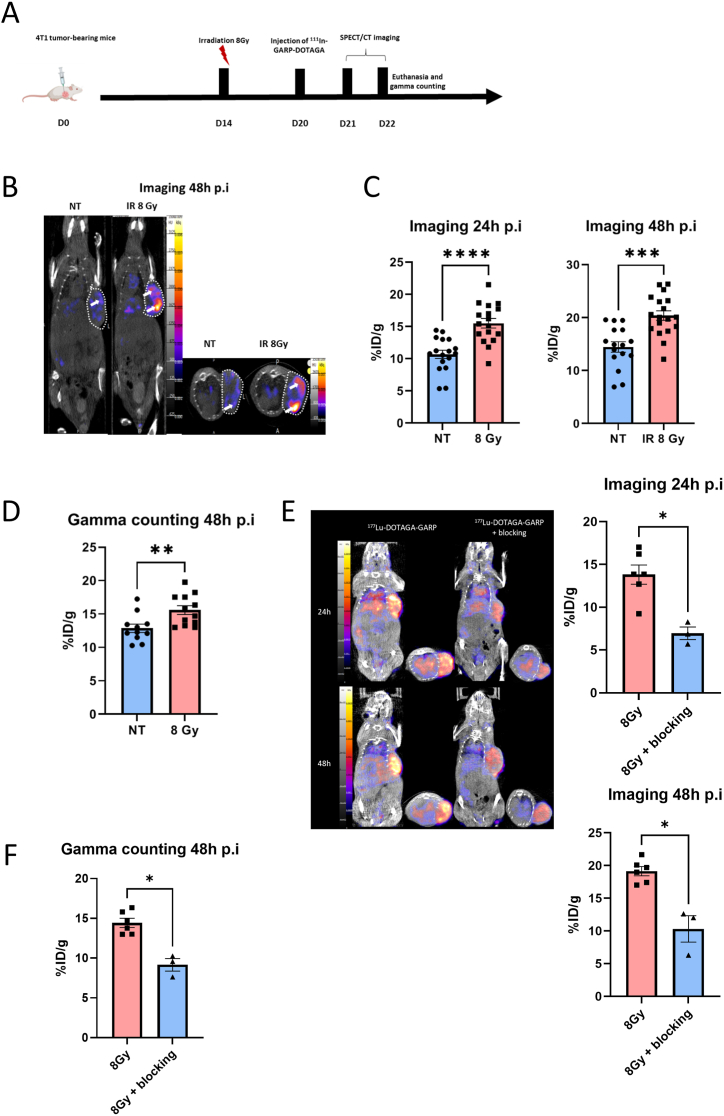
**External beam radiation therapy increases [**^**111**^**In]In-GARP-DOTAGA uptake in vivo by SPECT/CT imaging.** A. 14 days after 4T1 triple negative murine breast cancer, mice were assigned in 2 groups: external beam radiation at the dose of 8 Gy or control without irradiation. 7 days post irradiation, they underwent SPECT/CT imaging at 24h and 48h post-injection with [^111^In]In-GARP-DOTAGA. Mice were sacrificed after the last imaging and gamma counting was performed *ex vivo* on tumours. B. Representative SPECT/CT images of 4T1 tumour-bearing mice irradiated (n = 17) or not (n = 18) at 24h and 48h post-injection of 15MBq/100 μL of [^111^In]In-DOTAGA-GARP. Tumours are highlighted with circles. C. The scatter dot plot represents the percentage of the injected dose of [^111^In]In-DOTAGA-GARP per gram of tumour (%ID/g) of 4T1 tumour-bearing mice irradiated (n = 17) or not (n = 18) at 24h and 48h post-injection of 15 MBq/100 μL of [^111^In]In-DOTAGA-GARP. Results are presented as the median with the interquartile range, (∗∗∗∗p < 0.0001 ∗∗∗p = 0.0001). D. The scatter dot plot represents the percentage of injected dose of ^111^In-DOTAGA-GARP per gram of tumour of 4T1 tumour-bearing mice irradiated (n = 11) or not (n = 12) at 48h post-injection of 15 MBq/100 μL of [^111^In]In-GARP-DOTAGA measured by gamma counting. Results are presented as the median with the interquartile range, (∗∗p = 0.0056). E. Representative SPECT/CT images of 4T1 tumour-bearing mice irradiated (8Gy) at 24h and 48h post-injection of 15MBq/100 μL of [^111^In]In-DOTAGA-GARP (n = 6) or [^111^In]In-DOTAGA-GARP + 100x unlabelled DOTAGA-GARP (blocking, n = 3). The scatter dot plot represents the percentage of the injected dose of [^111^In]In-DOTAGA-GARP per gram of tumour (%ID/g) of 4T1 tumour-bearing mice irradiated (8Gy) at 24h and 48h post-injection of 15 MBq/100 μL of [^111^In]In-DOTAGA-GARP or blocking. Results are presented as the median with the interquartile range, (∗p < 0.05). F. The scatter dot plot represents the percentage of the injected dose of [^111^In]In-DOTAGA-GARP per gram of tumour (%ID/g) of 4T1 tumour-bearing mice irradiated (8Gy) at 48h post-injection of 15 MBq/100 μL of [^111^In]In-DOTAGA-GARP or blocking measured by gamma-counting. Results are presented as the median with the interquartile range, (∗p < 0.05).

4T1 tumour bearing mice were irradiated or not at the dose of 8 Gy at day 14 after implantation. 7 days and 8 days post-irradiation mice underwent SPECT/CT imaging at 24h and 48h post-injection with [^111^In]-DOTAGA-GARP (25 μg/15MBq/100 μL per mouse, Fig. 3A). At 24h and 48h post-injection, the tumour uptake of [^111^In]-DOTAGA-GARP was significantly higher in the irradiated group compared with non-irradiated mice (respectively 15.52 % ± 0.74 % ID/g *vs* 10.70 % ± 0.64 %ID/g, ∗∗∗∗p < 0.0001 at 24h post-irradiation and 20.38 % ± 0.90 % ID/g *vs* 14.45 % ± 0.99%ID/g, ∗∗∗p = 0.0001 at 48h post irradiation, Fig. 3C). Results were confirmed by *ex vivo* gamma counting at 48h post-injection (15.58 % ± 0.66 % ID/g *vs* 12.86 % ± 0.63 % ID/g, ∗∗p = 0.0056, Fig. 3D). In addition, the concomitant administration of [^111^In]In-DOTAGA-GARP with a 100x excess of unlabelled anti-GARP antibody induces a significant decrease in tumour uptake of [^111^In]In-DOTAGA-GARP thus demonstrating active targeting of our probe *in vivo* (Fig. 3E and F). To further confirm the active targeting of GARP^+^ Tregs by our probe *in vivo*, we demonstrate that the tumour uptake of [^111^In]In-DOTAGA-GARP is significantly decreased in 4T1 tumours in Nude mice lacking Tregs compared to Balb/C mice (Supplemental Fig. 4).

### 3.4 Targeted radionuclide therapy with [^177^Lu]Lu-DOTAGA-GARP limits tumour growth *in vivo*

**Fig. 4:**
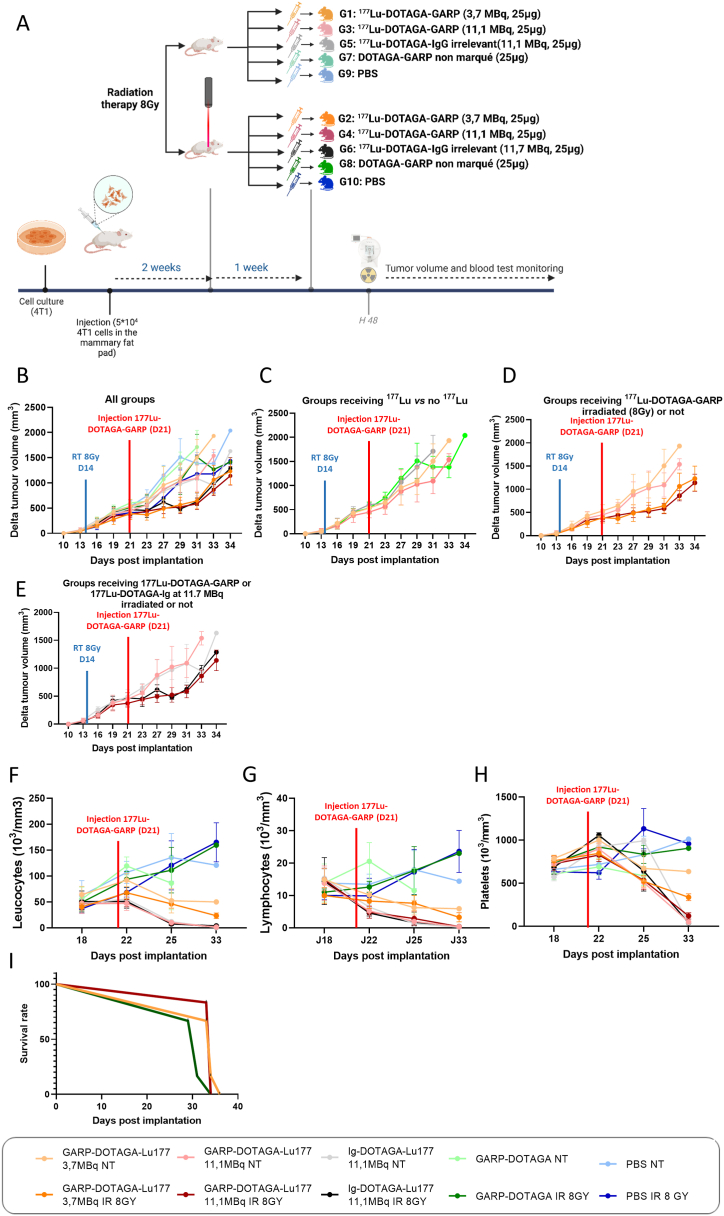
^**177**^**Lu-DOTAGA-GARP limits tumour growth.** A. Schematic representation of the experimental design: 4T1 tumour-bearing mice were randomized into 5 groups of 12 mice at 14 days post-implantation, mice in each group are subdivided in two groups receiving (IR n = 6) or not (NT n = 6) external radiation therapy at the dose of 8Gy 7 days post-irradiation, mice received: one i.v. dose of 25 μg/3,7 MBq/100 μL of [^177^Lu]Lu-DOTAGA-GARP per mouse (G1 = 6 NT and G2 = 6 IR 8Gy), one i.v. dose of 25 μg/11,7 MBq/100 μL of [^177^Lu]Lu-DOTAGA-GARP per mouse (G3 = 6 NT and G4 = 6 IR 8Gy), one i.v. dose of 25 μg/11,7 MBq/100 μL of [^177^Lu]Lu-DOTAGA-irrelevant antibody per mouse (G5 = 3 NT and G6 = 3 IR 8Gy), one i.v. dose of 25 μg/100 μL of non-radiolabelled DOTAGA-GARP per mouse (G7 = 6 NT and G8 = 6 IR 8Gy), one i.v. dose of 100 μL PBS per mouse(G9 = 6 NT and G10 = 6 IR 8Gy). B/C/D/E. 4T1 delta tumour growth (mm^3^) measured 3 times per week according to treatment group previously described. Results are presented as the median with the interquartile range. F/G/H. Total white blood cells (10^3^/mm^3^), lymphocytes (10^3^/mm^3^) and platelets (10^3^/mm^3^) measured 2 times per week according to treatment group previously described. Results are presented as the median with the interquartile rang. I. Kaplan-Meier survival plot for 4T1 tumour-bearing mice treated with treatment previously described.

Tumour volume of 4T1 tumour-bearing mice treated with a single i.v. dose of [^177^Lu]-DOTAGA-GARP (at a dose of 3.7MBq or 11.7MBq) or unlabelled-DOTAGA-GARP or vehicle (PBS) was measured three times a week (Fig. 4A). The [^177^Lu]-DOTAGA-GARP probe, regardless of the dose, induced a significant reduction in tumour growth from day 8 after injection compared to mice treated with PBS (tumour volumes: G1 [^177^Lu]Lu-DOTAGA-GARP 3.7MBq 1090.60 mm^3^ ± 123.73 mm^3^ tumour volume *vs* G9 PBS 1511.02 mm^3^ ± 149.80 mm^3^, ∗∗∗p = 0.0008; G3 [^177^Lu]Lu-DOTAGA-GARP 11.7MBq 1025.72 mm^3^ ± 149.76 mm^3^ tumour volume *vs* G9 PBS 1511.02 mm^3^ ± 149.80 mm^3^, ∗∗∗∗p < 0.0001, Fig. 4B). The [^177^Lu]Lu-DOTAGA-GARP probe, also induced a significant reduction in tumour growth from day 8 after injection compared to mice treated with the unlabelled-DOTAGA-GARP (tumour volumes: G3 ^177^Lu-DOTAGA-GARP 11.7MBq 1025.72 mm^3^ ± 149.76 mm^3^ *vs* G7 unlabelled DOTAGA-GARP 1382.32 mm^3^ ± 102.05 mm^3^, ∗p = 0.0104, Fig. 4C). The unlabelled-DOTAGA-GARP had no effect on tumour growth compared to mice treated with PBS (tumour volumes: G7 unlabelled DOTAGA-GARP 1382.32 mm^3^ ± 102.05 mm^3^ tumour volume *vs* G9 PBS 1511.02 mm^3^ ± 149.80 mm^3^, p = 0.8978, Fig. 4B).

External irradiation combined with [^177^Lu]Lu-DOTAGA-GARP probe irradiation induced a significant decrease in tumour growth compared to external irradiation alone from day 8 after [^177^Lu]Lu-DOTAGA-GARP probe injection (tumour volumes: G2 ^177^Lu-DOTAGA-GARP 3.7MBq irradiated 8 Gy 565.32 mm^3^ ± 61.50 mm^3^ *vs* G10 PBS irradiated 1030.98 mm^3^ ± 92.62 mm^3^, ∗∗∗∗p < 0,0001 and G4 [^177^Lu]Lu-DOTAGA-GARP 11.7MBq irradiated 8 Gy 254.42 mm^3^ ± 53.37 mm^3^ *vs* G10 PBS irradiated 1031.00 mm^3^ ± 92.62 mm^3^, ∗∗∗∗p < 0,0001 Fig. 4B). There was no significant difference in tumour growth between the groups treated with 3.7MBq or 11.7MBq of [^177^Lu]-DOTAGA-GARP (tumour volumes: G1 [^177^Lu]Lu-DOTAGA-GARP 3.7MBq non irradiated 958.10 mm^3^ ± 149.30 mm^3^ *vs* G3 [^177^Lu]Lu-DOTAGA-GARP 11.7MBq non irradiated 878.90 mm^3^ ± 114.36 mm^3^, p = 0.9943 and G2 [^177^Lu]Lu-DOTAGA-GARP 3.7MBq irradiated 8 Gy 486.63 mm^3^ ± 72.94 mm^3^ *vs* G4 [^177^Lu]Lu-DOTAGA-GARP 11.7MBq irradiated 8 Gy 495.90 mm^3^ ± 44.90 mm^3^, p > 0.9999 Fig. 4B).

In addition, the [^177^Lu]Lu-DOTAGA-GARP probe, regardless of the dose (3.7 and 11.7 MBq), induced a significant reduction in tumour growth from day 6 after injection when mice were treated by RT at the dose of 8 Gy 7 days before injection of the probe compared with non-irradiated mice (tumour volumes: G1 [^177^Lu]Lu-DOTAGA-GARP 3.7MBq non irradiated 958.10 mm^3^ ± 149.30 mm^3^ *vs* G2 [^177^Lu]Lu-DOTAGA-GARP 3.7MBq irradiated 8 Gy 486.63 mm^3^ ± 72.94 mm^3^, ∗∗∗∗p < 0,0001 and G3 [^177^Lu]Lu-DOTAGA-GARP 11.7MBq non irradiated 878.90 mm^3^ ± 114.36 mm^3^ *vs* G4 [^177^Lu]Lu-DOTAGA-GARP 11.7MBq irradiated 8 Gy 495.90 mm^3^ ± 44.90 mm^3^, ∗∗∗∗p < 0,0001, Fig. 4D). Of note, the radiation therapy induced a transient reduction in tumour growth significant from day 9 (tumour volumes: G9 PBS non irradiated 743.02 mm^3^ ± 49.26 mm^3^ *vs* G10 PBS irradiated 422.43 mm^3^ ± 29.14 mm^3^, ∗p = 0.0215) to day 17 (G9 PBS non irradiated 1383.60 mm^3^ ± 225.30 mm^3^ *vs* G10 PBS irradiated 1178.22 mm^3^ ± 153.62 mm^3^, p = 0.7308) after radiation therapy at the dose of 8 Gy.

The [^177^Lu]Lu-DOTAGA-GARP 11.7 MBq probe induced no significant difference on tumour growth compared to mice treated with the same activity of an irrelevant radiolabelled antibody – [ ^177^Lu]Lu-DOTAGA-Ig irrelevant (tumour volumes: G3 [^177^Lu]-DOTAGA-GARP 11.7MBq non irradiated 878.90 mm^3^ ± 114.36 mm^3^ tumour volume *vs* G5 [^177^Lu]Lu-DOTAGA-Ig irrelevant 11.7 MBq non irradiated 836.00 mm^3^ ± 184.15 mm^3^, p > 0.9999 and G4 [^177^Lu]Lu-DOTAGA-GARP 11.7MBq irradiated 8 Gy 495.90 mm^3^ ± 44.90 mm^3^ *vs* G6 [^177^Lu]Lu-DOTAGA-Ig irrelevant 11.7 MBq irradiated 8 Gy 616.50 mm^3^ ± 50.44 mm^3^ tumour volume, p = 0.9734, Fig. 4E).

However, in terms of haematotoxicity, the 3.7MBq dose of [^177^Lu]Lu-DOTAGA-GARP probe induced a lower decrease in white blood cells (Fig. 4F, G, H) and a longer survival (Fig. 4I) than the 11.7MBq dose while the number of animals remains too low to reach statistical significance.

Most interestingly, following external RT (8Gy), [^177^Lu]Lu-DOTAGA-GARP induced a significant decrease in Tregs and GARP^+^ Treg in tumours regardless of the dose compared with unlabelled-DOTAGA-GARP (Supplemental Fig. 5). Interestingly, [^177^Lu]Lu-DOTAGA-GARP also induced a significant decrease in Tregs and GARP^+^ Tregs in tumours compared with [^177^Lu]Lu-DOTAGA-Ig (Supplemental Fig. 5), thus confirming an active targeting of GARP by [^177^Lu]Lu-DOTAGA-GARP.

In addition, SPECT imaging of [^177^Lu]Lu-DOTAGA-GARP and [^177^Lu]Lu-DOTAGA-Ig 48h post-injection demonstrate a slight but significant increase in tumour uptake of [^177^Lu]Lu-DOTAGA-GARP compared to the isotype antibody (Supplemental Fig. 6).

## 4 Discussion

Radiation therapy is one of the most widely used treatments for a wide range of cancers (2). Effects of radiation therapy have been of considerable interest because of the possibility that therapy-induced changes in immune-cell homeostasis might interfere with anti-tumour activity notably by inducing Tregs invasion in the TME. GARP is highly expressed on activated Tregs and contributes to their immunosuppressive activity *via* the TGF-β axis (10, 24). GARP appears to be an attractive cancer target, due to its expression at the membrane of activated Tregs allowing their follow-up in the TME, its role in the function of Tregs improving their immunosuppressive activity and its expression in various cancer cells.

*Ex vivo* studies in our murine TNBC model indicated that RT increases GARP expression in tumours, both in cancer cells and Tregs. Post-RT Tregs, present in greater quantities within TME, have enhanced GARP expression at their membrane. These results are in accordance with Schuler et al. who found an increase in the number of Tregs and GARP expression at their membrane after chemoradiotherapy in head and neck squamous cell carcinoma patients [34]. The expression of GARP at the membrane of cancer cells, especially in bone sarcoma cancer cells, has also been shown to be responsible for resistance to radiotherapy [35]. In this context, the monitoring of this immunosuppressive receptor *in vivo* in the TME after RT could be of great importance.

Our study demonstrates that SPECT *in vivo* imaging using a GARP-targeted radiolabelled antibody allows to detect and quantify the increase in GARP ^+^ cells in a murine model of TNBC. While significant and in accordance with our *ex vivo* results, the tumour uptake of our probe after RT remains modest compared to control mice. These results highlight the complexity to image a small population of cells, especially immune cells. The relatively low uptake values observed in our study are in accordance with published results tracking immune cells such as CD8^+^ and CD206^+^ M2 like-macrophages with a %ID/g in tumour around 3 % [36,37] and 1 % [38] respectively. However, due to its exquisite sensitivity, SPECT/CT makes it possible to observe and quantify a low but significant variation in these cellular populations.

To implement a theranostic approach combining imaging and targeted radionuclide therapy (TRT), the DOTAGA-GARP antibody-based probe previously labelled with indium-111 (gamma emitter enabling detection by SPECT imaging) used for *in vivo* GARP detection by nuclear imaging with SPECT was then radiolabelled with lutetium-177 (beta minus emitter for targeted radionuclide therapy), enabling the specific irradiation of GARP^+^ cells. GARP is known to be expressed at the membrane of activated Tregs and cancer cells [20]. It has been shown that freshly isolated human Tregs express a low level of surface GARP which can be upregulated after T cell receptor stimulation [39] suggesting a targeting of GARP^+^ Tregs only in the tumour. This suggests that not all Tregs on the body are high expresser of GARP, leading to the rationale to develop this [^177^Lu]Lu-DOTAGA-GARP radiopharmaceutical candidate to target Tregs of the TME. Nevertheless, one cannot exclude the targeting of activated Tregs in other organs (*e.g.* intestine) by such a probe. The potential side effects of [^177^Lu]Lu-DOTAGA-GARP outside of the tumour may require further investigation.

In our murine TNBC model, the injection of [^177^Lu]Lu-DOTAGA-GARP induced a significant reduction in tumour growth from 8 days after injection compared to mice treated with vehicle (PBS) or unlabelled DOTAGA-GARP suggesting that the mechanism of action inhibiting tumour growth is mainly due to the radiation emitted by ^177^Lu. In addition, [^177^Lu]Lu-DOTAGA-GARP induced a significant reduction in tumour growth in mice receiving external RT while it had a lower impact on non-irradiated mice. These results are in line with our *in vivo* imaging results showing an increased uptake of [^111^In]In-DOTAGA-GARP in the tumour after RT suggesting a higher expression of GARP. Thus, an increased uptake of [^177^Lu]Lu-DOTAGA-GARP led to a higher irradiation within the tumour with a subsequent reduced tumour growth. These results further validate our theranostic strategy demonstrating that SPECT imaging with the GARP targeted probe may be a powerful tool to foresee its potential therapeutic effect. This strategy has been associated with recent clinical successes such as [^177^Lu]Lu-PSMA617 and [^177^Lu]Lu-oxodotreotide for prostate and neuroendocrine tumours respectively. In the case of treatment with [^177^Lu]Lu-PSMA617, diagnostic PET imaging is performed with [^68^Ga]Ga-PSMA11 (as a companion diagnostic) to select patients whose tumours overexpress this receptor. Several studies have investigated the relationship between pre-treatment uptake of the companion diagnostic and response to treatment with [^177^Lu]Lu-PSMA617, showing its value in predicting response to treatment, as well as the dose reaching the tumour and healthy tissue in order to limit side effects [[40], [41], [42]]. Theranostic approach targeting PSMA has been shown to be effective in prostate cancer and has recently been approved [43,44]. In breast cancer, similar strategies are currently under clinical investigations with imaging assessment of PSMA expression (NCT06059469) and theranostic strategies targeting HER2 (NCT04674722).

In our study two doses of [^177^Lu]-DOTAGA-GARP were tested: 3.7MBq and 11.7MBq. While efficacy on tumour growth showed no significant difference between the two doses, the highest dose showed a higher haematological toxicity and a lower survival rate. Haematological toxicity is a well-recognized complication of TRT with lutetium-labelled vectors with a long circulation time [45,46]. In our study, the DOTAGA-GARP probe that we generated was based on radiolabelling of a whole-length monoclonal antibody characterized by a long blood circulation time. Whole antibodies, which have a large molecular size (150 kDa), circulate in the blood for 3–4 weeks after injection. This prolonged plasma half-life is the cause of dose-limiting radiotoxicity to non-target organs and poor diffusion rates. The use of monoclonal antibody fragments of a smaller size, such as, Fab (50 kDa) or Fab'2 (100 kDa) may improve tumour uptake and may limit toxicity [47,48]. Antibody fragments show a more rapid and better tumour penetration, homogeneous distribution and can bind to cryptic epitopes that are unreachable by full-size antibody [49]. However, these fragments are characterized by a more rapid progression towards the necrotic centre, potentially decreasing the absorbed dose to the outer viable tumour region. They also have a faster blood clearance time and rapid excretion (largely through the kidneys) within 2–4h, thereby reducing the tumour uptake and possibly leading to renal radiotoxicity [50,51]. Finally, small molecules have better tumour-to-non target tissue uptake, but at the cost of lower absolute uptake in the tumour. Nevertheless, in clinical practice, even the use of antibody fragments was not always accompanied by the expected success [47]. Whether antibody fragments are more favourable for TRT purposes remains an open question that needs further investigation.

Finally, the efficacy of our [^177^Lu]Lu-DOTAGA-GARP was also compared with an irrelevant antibody labelled with lutetium-177 ([^177^Lu]Lu-DOTAGA-Ig irrelevant) at a similar dose (11.7MBq). Unfortunately, there was no significant difference in tumour growth between mice receiving [^177^Lu]Lu-DOTAGA-GARP or [^177^Lu]Lu-DOTAGA-Ig irrelevant. This finding can be attributed to the enhanced permeability and retention (EPR) effect leading to a passive tumour uptake of [^177^Lu]Lu-DOTAGA-Ig and subsequently an unspecific irradiation of tumour cells. It is well known that 4T1 tumour is an EPR-positive tumour model [[52], [53], [54]]. Consequently, two mechanisms enable the [^177^Lu]Lu-DOTAGA-GARP probe to reach and irradiate cells in the tumour in our model: active targeting *via* the anti-GARP monoclonal antibody and passive targeting *via* the EPR effect. We demonstrate here by immunostaining and cytometry that total GARP expression within the tumour is relatively low (<5 %), suggesting a low proportion of active targeting masked by passive targeting, which may explain why there was no difference in efficacy on tumour growth between the [^177^Lu]Lu-DOTAGA-GARP probe and the [^177^Lu]Lu-DOTAGA-Ig irrelevant probe. Despite clear proof that our probe is selectively targeting GARP^+^ cells *in vivo*, our results demonstrate only a very slight increase in tumour uptake of the GARP targeted probe compared to the isotype control thus confirming that GARP targeting may not be sufficient to outperform the EPR effect. While this effect has been widely described in rodent models, its efficacy in human tumours remains controversial with most EPR-driven drugs falling to translate into the clinic [55,56]. In clinic, tumour targeting *via* the ERP effect has shown high heterogeneity among patients depending on tumour localization, tumour type and tumour microenvironment (hypoxic/immune status) [57]. As passively targeted drugs dependent on the EPR effect may not be sufficient to achieve effective doses at target sites [58], the active targeting of GARP was evaluated in our study. However, the low level of GARP expression in tumours did not allow a sufficient increase in effective doses at the target site to further promote an anti-tumour efficacy beyond the EPR effect. The results of Kang et al. correlated with our findings, showing no difference in tumour growth between their group receiving the [^177^Lu]Lu-CD38 probe and [^177^Lu]Lu-Ig irrelevant in a low CD38^+^ expressing model due to the similar tumour uptake between the two groups. In a high CD38^+^ expressing model, the authors were able to demonstrate a significantly increased anti-tumour effect with the [^177^Lu]Lu-CD38 compared to the [^177^Lu]Lu-Ig irrelevant [59]. These findings highlight that the effective therapy was closely related to its tumour uptake and suggested that the DOTAGA-GARP probe could be tested in other preclinical models with higher GARP expression.

Currently, several therapeutic antibodies against GARP are under evaluation in clinical trials (*e.g.* phase 1: NCT04419532, NCT05606380, NCT05821595, NCT05869240 in unspecific advanced solid tumours or lymphoma and phase 2/3: NCT06236438 in untreated metastatic non-squamous non-small cell lung cancer (NSCLC)) [25,60,61]. De Streel *at al*. demonstrated that their anti-GARP antibody did not display anti-tumour activity when administered as a monotherapy alone in a CT26 tumour mouse model suggesting that the amount of GARP in the tumour may not be sufficient to induce tumour regression. However, in combination with immunotherapy (anti-PD-1), this antibody improved its efficacy [60]. In our TNBC model, GARP expression was likely too low to show an efficient GARP-targeted TRT treatment, but it could also be used to improve current immunotherapies as suggested by recent findings demonstrating that low-dose TRT may render immunologically cold tumours responsive to immune checkpoint blockade [62]. In addition, the recent rise in the use of ADCs for breast cancer patients, including TNBC, highlights the importance of developing combination strategies, in particular with TRT, especially in a context in which ADCs are only showing efficacy in a limited number of patients and in which resistance phenomena are emerging. Finally, the identification of TME-specific targets (*e.g.* GARP for activated Tregs) may offer targets that are more universally expressed since the TME composition may be more conserved among cancer types and TME-specific antigens may be less genetically unstable than tumour antigens on cancer cells [63]. Our study demonstrates that a GARP targeted theranostic probe may be an innovative strategy to detect and quantify GARP expression *in vivo* in our TNBC model in order to perform tumour phenotyping, determine aggressiveness and predict escape or resistance to treatments. In view of the model's limitations in terms of GARP expression and EPR effect, this probe should be further tested in other preclinical cancer models. Finally, this GARP targeted theranostic probe may be a relevant strategy to detect patients with high tumour expression of GARP (*e.g.* following RT) in order to select them for anti-GARP therapies.

## CRediT authorship contribution statement

**Pierre-Simon Bellaye:** Writing – review & editing, Writing – original draft, Supervision, Methodology, Investigation, Conceptualization. **Alexandre MM. Dias:** Writing – review & editing, Methodology. **Jean-Marc Vrigneaud:** Writing – review & editing, Visualization, Methodology. **Alexanne Bouchard:** Writing – review & editing, Methodology. **Mathieu Moreau:** Writing – review & editing, Methodology. **Camille Petitot:** Writing – review & editing, Methodology. **Claire Bernhard:** Writing – review & editing, Methodology. **Michael Claron:** Writing – review & editing, Methodology. **Lisa Froidurot:** Writing – review & editing, Methodology. **Véronique Morgand:** Writing – review & editing, Methodology. **Mélanie Guillemin:** Writing – review & editing, Methodology. **Marie Monterrat:** Writing – review & editing, Methodology. **Céline Mirjolet:** Writing – review & editing, Visualization, Methodology. **Carmen Garrido:** Writing – review & editing, Supervision. **Evelyne Kohli:** Writing – review & editing, Writing – original draft, Supervision, Funding acquisition, Conceptualization. **Bertrand Collin:** Writing – review & editing, Visualization, Supervision, Funding acquisition, Conceptualization.

## Data availability

The datasets generated during and/or analysed during the current study are available from the corresponding author on reasonable request.

## Ethics approval

All animal studies were performed in compliance with preclinical ethical guidelines and approved by the local ethical committee (C2EA Grand Campus, project #34968, Dijon) and the French ministry of Research.

## Declaration of competing interest

The authors declare that they have no known competing financial interests or personal relationships that could have appeared to influence the work reported in this paper.
