# Supplementary material for: Targeted radionuclide therapy against GARP expressing T regulatory cells after tumour priming with external beam radiotherapy in a murine syngeneic model
Source: Heliyon. 2024 Oct 18;10(20):e39543. doi: 10.1016/j.heliyon.2024.e39543 (PMC11533616; doi:10.1016/j.heliyon.2024.e39543)
Supplement: Multimedia component 1 [file mmc1.pdf]

Targeted radionuclide therapy against GARP expressing T regulatory cells after tumour priming with external beam radiotherapy in a murine syngeneic model

Pierre-Simon Bellaye[1], Alexandre MM Dias[1], Jean-Marc Vrigneaud[1], Alexanne Bouchard[1,2], Mathieu Moreau[3], Camille Petitot[1], Claire Bernhard[3], Michael Claron[3], Lisa Froidurot[1], Véronique Morgand[1], Mélanie Guillemin[1], Marie Monterrat[1], Céline Mirjolet[1], Carmen Garrido[2], Evelyne Kohli[2,4] and Bertrand Collin[1,3]*.

Affiliations:

[1] Centre George-François Leclerc, Service de Médecine Nucléaire, IMATHERA UMS INSERM BioSanD US58, 1 rue du Professeur Marion, 21079 Dijon, France

[2]UMR INSERM/uB/AGROSUP 1231, Labex LipSTIC, Faculty of Health Sciences, Université de Bourgogne Franche-Comté, 21079 Dijon, France

[3] Institut de Chimie Moléculaire de l'Université de Bourgogne, UMR CNRS/uB 6302, Université de Bourgogne Franche-Comté, 21079 Dijon, France

[4] University Hospital Centre François Mitterrand, 21000 Dijon, France

*Author to whom correspondence should be addressed.

Corresponding author: bertrand.collin@u-bourgogne.fr

Supplementary material

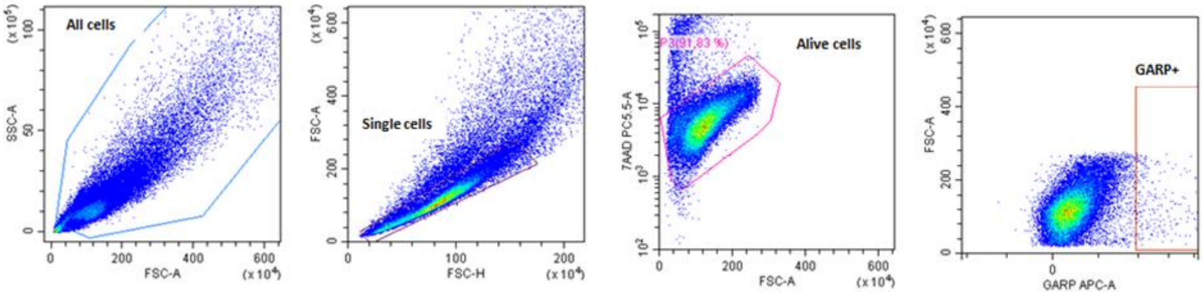

Supplementary Figure 1: Gating strategy for GARP+ in vitro detection on 4T1 cells.

A

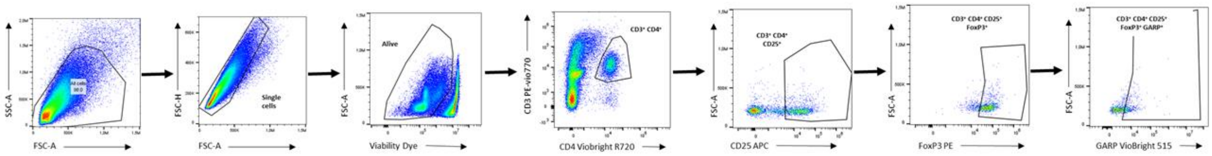

B

Marker	Fluorochrome	Clone
**CD3**	PE-Vio770	REA606
**CD4**	VioBright R720	REA604
**CD25**	APC	RE568
**FoxP3**	PE	REA788
**GARP**	Vio-Bright 515	REA139
**Viability**	PB450	L34964

Supplementary Figure 2: A. Gating strategy for regulatory T cells GARP+ identification in tumour tissue. B. List of antibodies used for identification of regulatory T cells GARP+ identification in tumour tissue.

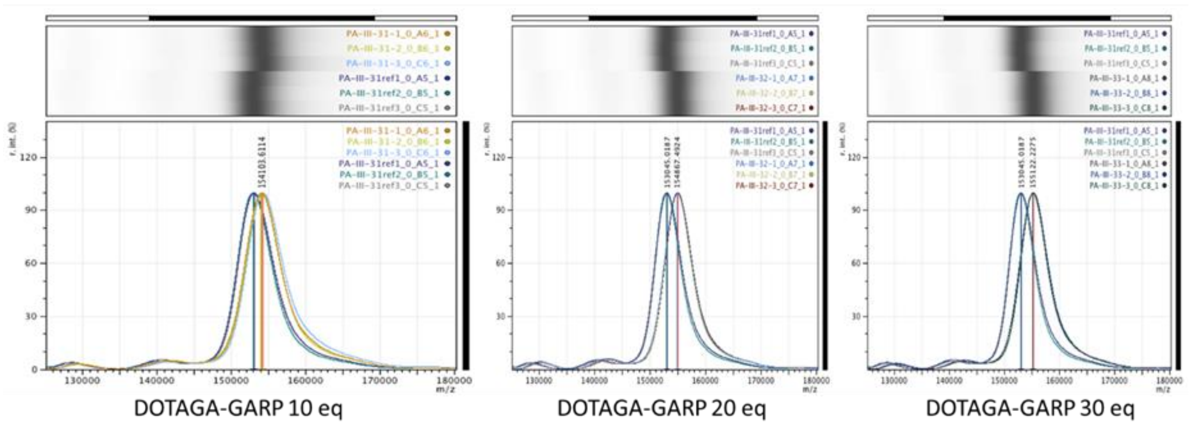

Supplementary Figure 3: The degrees of labelling (DOL) were determined by mass spectrometry (MALDI-TOF) and were 2.5, 4.1 and 4.7 for 10, 20 and 30 equivalents, respectively.

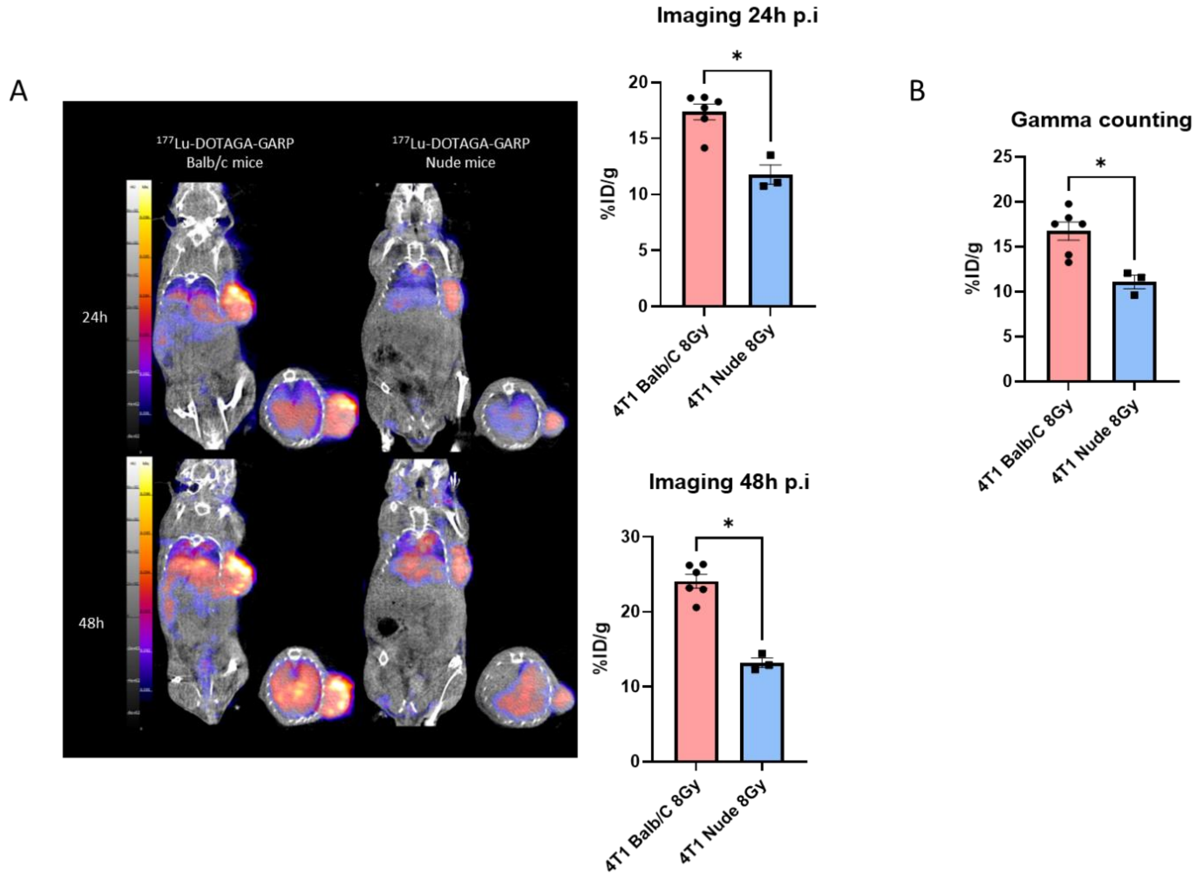

*Supplementary Figure 4: A. Representative SPECT/CT images of 4T1 tumour-bearing mice irradiated (8Gy) at 24h and 48h post-injection of 15MBq/100 µL of [^{111}In]In-DOTAGA-GARP in Nude (n=3) or Balb/C (n=6) mice. The scatter dot plot represents the percentage of the injected dose of [^{111}In]In-DOTAGA-GARP per gram of tumour (%ID/g) of 4T1 tumour-bearing mice irradiated (8Gy) at 24h and 48h post-injection of 15 MBq/100 µL of [^{111}In]In-DOTAGA-GARP in Nude (n=3) or Balb/C (n=6) mice. Results are presented as the median with the interquartile range, (*p<0.05). F. The scatter dot plot represents the percentage of the injected dose of [^{111}In]In-DOTAGA-GARP per gram of tumour (%ID/g) of 4T1 tumour-bearing mice irradiated (8Gy) at 48h post-injection of 15 MBq/100 µL of [^{111}In]In-DOTAGA-GARP in Nude (n=3) or Balb/C (n=6) mice measured by gamma-counting. Results are presented as the median with the interquartile range, (*p<0.05).*

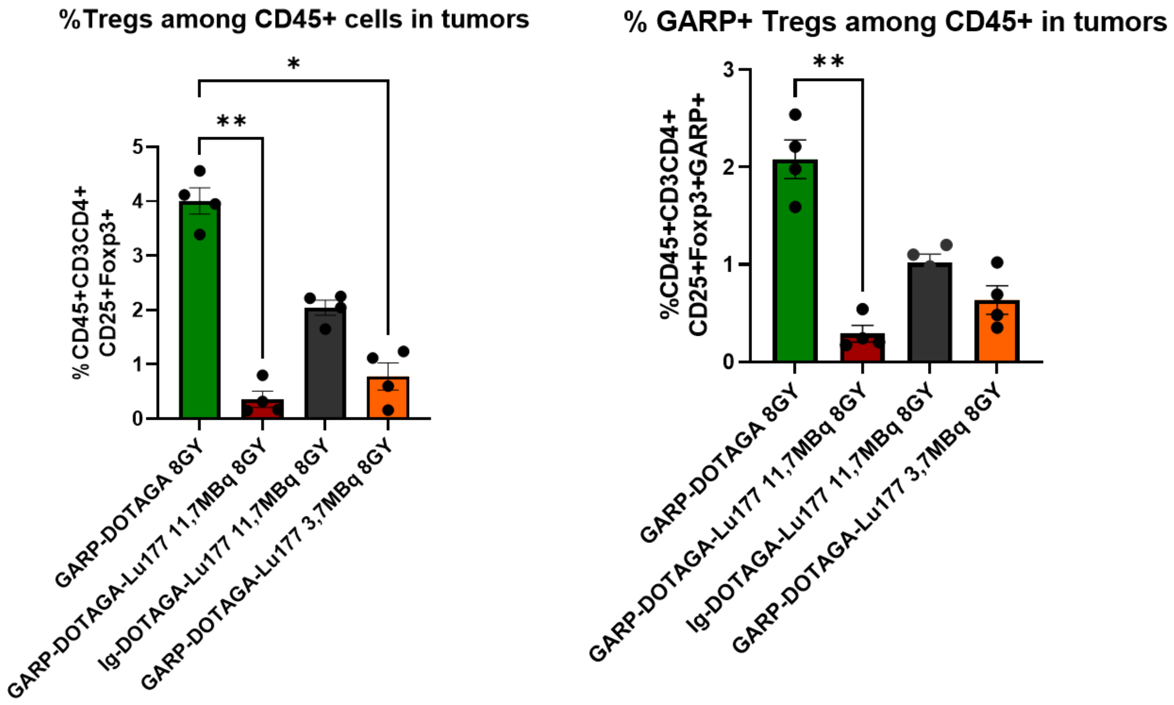

Supplementary Figure 5: Flow cytometry of percent of Tregs (left panel) and GARP+ Tregs (right panel) in 4T1 tumours following RT (8Gy) and i.v. injection of [^{177}Lu]Lu-DOTAGA-GARP (11.7MBq or 3.7 MBq) or ^{177}Lu-DOTAGA-Ig.

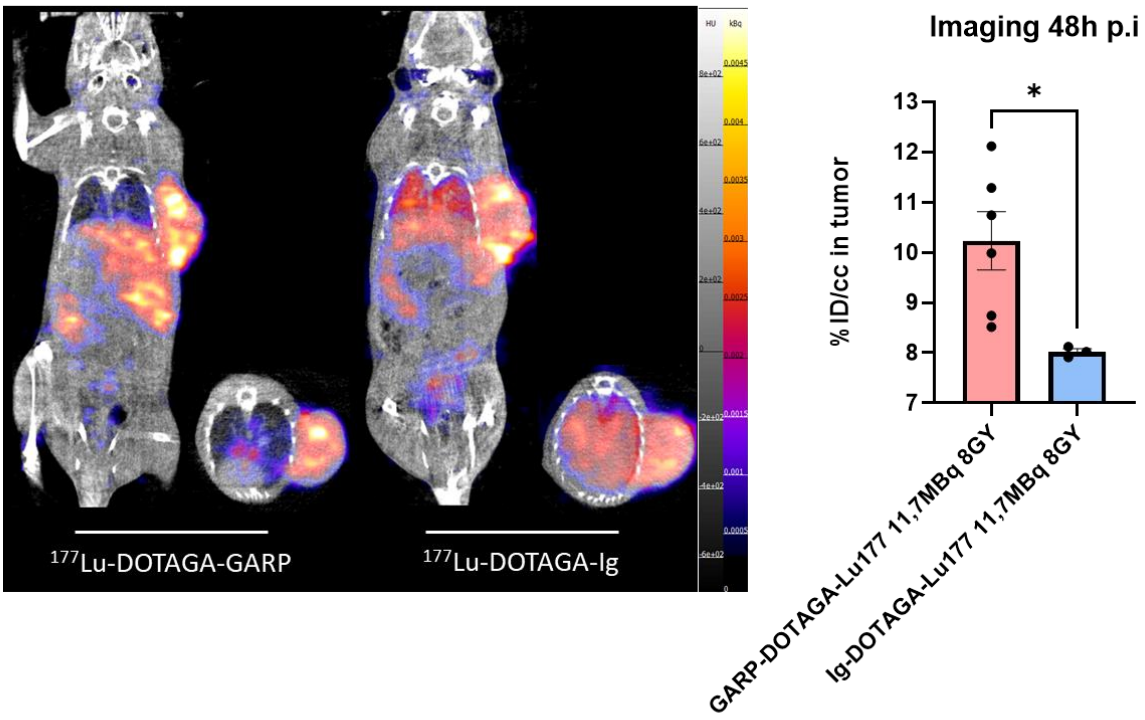

Supplementary Figure 6: Representative SPECT/CT imaging and tumour uptake quantification of [^{177}Lu]Lu-DOTAGA-GARP and [^{177}Lu]Lu-DOTAGA-Ig 48h post-injection.
